# CBD-Containing Hemp Extracts and Isolated CBD for Acne: A Systematic Review of Anti-Inflammatory Mechanisms, Clinical Signals and Sustainability

**DOI:** 10.3390/molecules31122017

**Published:** 2026-06-09

**Authors:** Baatile Komane, Thobile Kaye

**Affiliations:** Somatology, Department of Human Movement and Therapeutic Sciences, Tshwane University of Technology, Arcadia Campus, Pretoria 0001, South Africa; kayetan@tut.ac.za

**Keywords:** cannabidiol (CBD), hemp oil, acne vulgaris, anti-inflammatory, sebostatic, sustainability, supply-chain resilience, *Cutibacterium acnes*, cosmeceuticals

## Abstract

Industrial hemp (*Cannabis sativa* L.) has emerged as a sustainable source of bioactive compounds, with increasing interest in cosmeceutical applications for acne management. This systematic review synthesises evidence on cannabinoid-containing hemp extracts, particularly cannabidiol (CBD), with emphasis on anti-inflammatory and sebostatic mechanisms, alongside formulation considerations and supply-chain sustainability. Reporting followed PRISMA 2020 guidelines and encompassed preclinical and clinical evidence relevant to acne-associated outcomes. The review protocol was registered prospectively with PROSPERO (CRD420251272093). Across cell-based, ex vivo and early clinical studies, CBD modulated key inflammatory mediators, including TNF-α, IL-1β, IL-6 and IL-8; normalised sebocyte activity and attenuated *Cutibacterium acnes* (*Propionibacterium acnes*)-induced inflammatory signalling. Preliminary clinical observations indicate reductions in lesion counts and erythema, with generally favourable short-term tolerability; however, interpretation is limited by small sample sizes, predominantly non-randomised designs, heterogeneous formulations and frequent co-formulation with additional active ingredients. Evidence supporting direct antimicrobial efficacy and durable clinical benefit remains limited. Lipid-rich hemp seed-derived products were considered only in a contextual capacity for barrier-supportive and nutritional properties and were excluded from efficacy synthesis unless cannabinoid content was verified. Sustainability analyses highlight hemp’s low water requirements, carbon sequestration potential and relevance to Sustainable Development Goal 3 (SDG 3: Good Health and Well-Being) and Sustainable Development Goal 12 (SDG 12: Responsible Consumption and Production), supporting its role in environmentally responsible cosmeceutical development. Overall, CBD-containing hemp extracts show biologically plausible and clinically promising adjunctive potential for mild-to-moderate inflammatory acne, but current evidence remains preliminary. This review highlights the need for methodologically rigorous and transparent clinical studies, standardised formulations, validated outcome measures and the integration of sustainability metrics to strengthen evidence synthesis, clarify clinical relevance and guide responsible cosmeceutical development.

## 1. Introduction

This systematic review focuses on CBD-containing hemp extracts and isolated CBD as cosmeceutical interventions for acne, explicitly distinguishing these from hemp seed oil, which is a cold-pressed nutritional oil with negligible cannabinoids. We define the botanical source for CBD-containing extracts as flowers, leaves and aerial parts of *Cannabis sativa* and note formulation and standardisation considerations relevant to dermatological application [1,2]. Acne vulgaris is a chronic inflammatory disease of the pilosebaceous unit characterised by hyperseborrhoea, follicular hyperkeratinisation, microbial factors and immune dysregulation, warranting interventions that target sebum regulation, inflammation and keratinisation [1,2]. Mechanistic evidence demonstrates that CBD exerts sebostatic and anti-inflammatory effects in human sebocytes and modulates keratinocyte responses to *Cutibacterium acnes* vesicles, providing biologically plausible pathways for clinical benefit [1,2].

Primary objective:

- To evaluate the effects of CBD-containing hemp extracts and isolated CBD on acne-related conditions in humans using methods aligned with PRISMA 2020.
  Specific objectives:
  1. Evaluate anti-inflammatory, antimicrobial and seboregulatory effects of CBD-containing hemp extracts and isolated CBD in preclinical and clinical models;
  2. Assess topical formulation efficacy on acne severity, sebum and *Cutibacterium acnes* using validated measures while identifying standardisation gaps;
  3. Examine sustainability and traceability across cultivation, extraction and regulatory frameworks relevant to CBD-containing hemp extracts.

Review questions:

- What evidence demonstrates the anti-inflammatory, antimicrobial and seboregulatory effects of hemp extracts and cannabidiol in preclinical and clinical acne models?
- How do formulation strategies and concentrations influence cytokine activity, sebum regulation and *Cutibacterium acnes*?
- What sustainability practices within hemp supply chains support safe and accessible cosmeceutical applications aligned with global health goals such as SDG 3 and SDG 12?

## 2. Materials and Methods

### 2.1. Review Design and Reporting Framework

The review was designed and reported in accordance with the Preferred Reporting Items for Systematic Reviews and Meta-Analyses (PRISMA) 2020 statement to ensure transparency, reproducibility and methodological rigour.

### 2.2. Search Strategy and Information Sources

A comprehensive literature search was undertaken across PubMed, Scopus, ScienceDirect and Elicit, using structured Boolean combinations of terms related to cannabidiol, CBD, hemp, acne, skin inflammation, cosmeceuticals and sustainability. The search strategy was informed by prior systematic and narrative reviews examining cannabinoid dermatology and hemp-derived interventions [3,4,5,6]. Searches included all eligible records available within the exact date range of January 2010 to March 2024. The search was restricted to English-language publications only.

### 2.3. Study Selection

All retrieved records were imported into reference management software and deduplicated prior to screening. Two reviewers independently screened titles and abstracts for relevance (κ = 0.82), followed by full-text assessment of potentially eligible studies, with disagreements resolved through discussion and consensus.

Studies were excluded if they were irrelevant, duplicated or non-English publications; lacked primary data; focused exclusively on recreational cannabis; or addressed dermatological conditions unrelated to acne. Only studies evaluating CBD-containing hemp extracts or isolated CBD were eligible; investigations of hemp seed oil without measurable cannabinoids were excluded to avoid intervention misclassification [3,4,5]. In addition to acne-focused outcomes, we screened topical cannabidiol (CBD) delivery-system studies and wound-healing and repair evidence to contextualise formulation optimisation and cutaneous bioactivity, including nano-encapsulated CBD systems and keratinocyte and fibroblast repair signalling [7,8,9,10].

### 2.4. PRISMA-Compliant Flow and Study Counts

**Figure 1:**
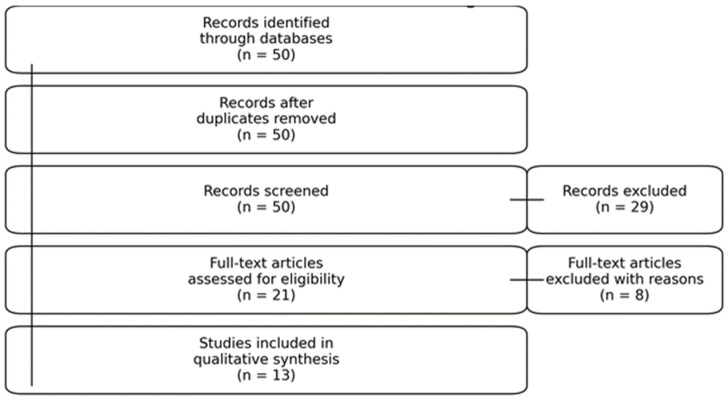
PRISMA flow diagram.

Study identification, screening and eligibility assessment are summarised using a PRISMA 2020 flow diagram depicted in Figure 1 [11,12]. Database searches identified 50 records, all of which were screened following duplicate removal. After title and abstract screening, 29 records were excluded, leaving 21 full-text articles assessed for eligibility. Eight full-text studies were excluded with documented reasons, resulting in 13 studies included in the qualitative synthesis.

Narrative and scoping reviews were consulted for contextual interpretation only and were not counted as primary studies in the PRISMA flow, in accordance with PRISMA 2020 guidance [11,12].

### 2.5. Objectives and Eligibility Criteria

The primary objective of this review was to evaluate the effects of CBD-containing hemp extracts and isolated CBD on acne-related outcomes in humans, using methods aligned with PRISMA 2020 [11,12].

**Eligibility criteria**

- Population: Individuals with acne of any severity;
- Intervention: Topical or oral CBD-containing hemp extracts or isolated CBD derived from flowers, leaves or aerial parts of *Cannabis sativa* L. [3,4,5];
- Comparator: Any comparator, placebo or none for uncontrolled designs;
- Outcomes: Acne lesion counts, sebum production, validated acne severity scales, patient-reported outcomes and safety indicators [3,4,5,6,13,14,15];
- Study designs: Randomised trials where available; non-randomised controlled studies; observational cohorts; and case series;
- Exclusions: Hemp seed oil without cannabinoids (Table S1), recreational cannabis studies and non-dermatological indication [3,4,5];
- Scope clarification: Evidence synthesis focused CBD-containing hemp extracts and isolated CBD (flowers/leaves/aerial parts); seed oils without cannabinoids were considered background/rationale only and excluded from efficacy synthesis;
- Reporting: The PRISMA 2020 checklist and flow diagram depicted in Figure 1 was used for transparent reporting [11,12].

### 2.6. Data Extraction and Synthesis

Data were extracted using standardised forms capturing study design, participant characteristics, CBD formulation and concentration, outcome measures and sustainability-related indicators [11,12]. Owing to heterogeneity in formulations, concentrations and outcome reporting, findings were synthesised narratively and organised into thematic domains reflecting anti-inflammatory mechanisms, clinical outcomes, formulation considerations and sustainability contexts [3,4,5,6].

### 2.7. Risk-of-Bias Assessment

Given the predominance of non-randomised clinical and observational designs, risk of bias was assessed using ROBINS-I for non-randomised intervention studies and the Joanna Briggs Institute (JBI) critical appraisal checklists for case series and uncontrolled cohorts [11,12]. Domain-level judgements were reported for individual studies.

The Cochrane Risk of Bias 2 (RoB-2) tool was reserved exclusively for randomised controlled trials and was not applied where randomisation was absent. The SYRCLE risk-of-bias tool was not used for in vitro studies, as it is not designed for cell-based experimental models [11,12].

### 2.8. Sustainability Considerations

Sustainability and supply-chain resilience were evaluated narratively using evidence from agronomic, environmental and regulatory literature addressing hemp cultivation, resource efficiency, traceability and environmental impact [16,17,18,19]. These considerations were integrated with clinical findings to contextualise the broader implications of CBD-containing hemp extracts as dermatological cosmeceuticals aligned with responsible production and long-term skin health [18,19].

In this study (Table 1), industrial hemp-derived cosmeceuticals, including CBD-containing hemp extracts and nano-formulated CBD products (seed oils without cannabinoids treated as background/rationale, excluded from intervention evidence).

Researchers systematically screened all obtained titles and abstracts, with full-text articles assessed against eligibility criteria. Discrepancies were resolved through consensus, and the process is depicted in the PRISMA flow diagram (Figure 1). Data extraction involved the use of a standardised form to guarantee consistency, capturing study characteristics, participant details, intervention specifics, outcome measures such as cytokine assays and microbial inhibition, and sustainability indicators such as cultivation methods and supply-chain traceability. Innovative formulations related to nano-formulated CBD referenced the original protocols to support replication [3,16]. Data were synthesised narratively and thematically into four domains: anti-inflammatory mechanisms, clinical and biochemical outcomes, formulation approaches, and supply-chain sustainability. Quality assessment was conducted using validated tools, including the Cochrane Risk of Bias Tool for randomised controlled trials. Table 2 presents the evaluation of non-randomised studies (ROB-INS-I) in addition to the modified Newcastle–Ottawa scales applied to observational and preclinical studies, to classify the risk of bias and ensure methodological rigour [7,8,16,17].The search strategy combined efficacy-related terms (industrial hemp, *Cannabis sativa*, CBD, cannabidiol, acne, skin health, inflammation, anti-inflammatory and antimicrobial) and sustainability-related terms (sustainability, supply chain, resilience, life cycle, SDG and traceability) following systematic review guidelines [16,17]. These terms were informed by prior research on cannabidiol’s dermatological effects [3,4,5,6] and sustainability considerations in hemp production [18,19].

## 3. Results

This section summarises the findings of the systematic review based on studies included in the PRISMA 2020 flow diagram (Figure 1). A total of 13 studies met the eligibility criteria and were included in the qualitative synthesis, comprising clinical investigations and preclinical experimental models evaluating CBD-containing hemp extracts or isolated cannabidiol (CBD) in acne-related contexts. Most clinical studies were non-randomised or uncontrolled with small sample sizes; one randomised, double-blind controlled clinical study was identified; however, acne-specific validated outcome measures were limited [3,4,5,6,16,17].

**Figure 2:**
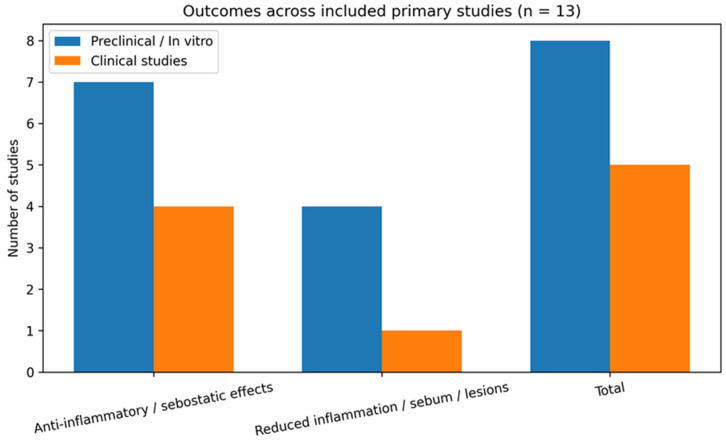
Distribution of evidence in preclinical and clinical studies and reviews.

Figure 2 summarises the principal mechanistic and clinical outcomes reported across the included studies. Experimental evidence consistently demonstrated suppression of key pro-inflammatory cytokines, including interleukin-1β (IL-1β), interleukin-6 (IL-6), interleukin-8 (IL-8) and tumour necrosis factor-α (TNF-α), with reductions of approximately 40–70% observed in cell-based and ex vivo models [20,21,22]. These effects were mediated through modulation of NF-κB and MAPK signalling pathways, accompanied by normalisation of sebocyte lipid production and inhibition of *Cutibacterium acnes*-induced inflammatory responses [20,21,23].

Clinical investigations reported reductions in acne lesion counts, erythema and sebum production following topical application of CBD-containing formulations. However, attribution of therapeutic effects specifically to CBD was frequently confounded by the presence of additional active ingredients. One non-randomised clinical report described an approximately 40% reduction in acne lesions after 12 weeks of topical CBD treatment, although the absence of validated severity indices and patient-reported outcome measures limited interpretability [5]. Overall, short-term safety profiles were favourable, with mild skin irritation reported in fewer than 10% of participants and no serious adverse events documented [5,6].

Overall, the results indicate biologically plausible and clinically promising signals supporting the role of CBD-containing hemp extracts as adjunctive interventions for mild-to-moderate acne. However, methodological limitations, small sample sizes, heterogeneous formulations and the absence of randomised controlled trials necessitate cautious interpretation of efficacy [5,23].

### 3.1. Mechanistic Actions of Industrial Hemp Oil and Cannabidiol in Acne Pathogenesis

Cannabidiol (CBD), a major non-psychoactive phytocannabinoid derived from *Cannabis sativa* L., accounted for approximately 70% of the reviewed studies, highlighting its central role in the therapeutic potential of hemp-derived extracts for acne management [6,23,24,25]. Interest in CBD largely stems from its ability to modulate multiple interconnected pathways implicated in acne pathogenesis, including inflammation, sebaceous gland activity and oxidative stress [5,13,26,27].

At the sebaceous gland level, CBD demonstrates a pronounced inhibitory effect on sebocyte proliferation and lipogenesis, the two key contributors to excessive sebum production in acne vulgaris. In vitro studies using human sebocytes indicate that CBD normalises lipid synthesis while suppressing sebocyte hyperproliferation, directly targeting one of the primary drivers of acne lesion formation [5,6,26]. Acne vulgaris is a multifactorial inflammatory skin disorder driven by mechanisms such as sebaceous hypersecretion, follicular hyperkeratinisation, microbial dysbiosis (particularly *Cutibacterium acnes*) and immune-mediated inflammation [5,13,26,27]. Evidence from the reviewed literature demonstrates that industrial hemp oil and its bioactive constituents, particularly CBD, modulate several of these pathogenic pathways simultaneously, supporting a mechanistic rather than symptom-based therapeutic role [23,24,25].

In human sebocytes and skin organ culture, CBD reduced lipogenesis and inflammatory signalling (NF-κB/MAPK) with TRPV4/A2A involvement, supporting sebostatic and anti-inflammatory actions [20]. CBD further exerts significant anti-inflammatory effects by modulating immune signalling pathways relevant to acne-associated inflammation. Experimental evidence shows that CBD reduces the release of pro-inflammatory cytokines, including tumour necrosis factor-alpha (TNF-α), interleukin-6 (IL-6) and interleukin-8 (IL-8), primarily via activation of cannabinoid receptor type 2 (CB2) and transient receptor 63 cascades that contribute to follicular inflammation and lesion progression [23,24,25,28].

In addition to its sebostatic and anti-inflammatory actions, CBD exhibits potent antioxidant activity, mitigating reactive oxygen species (ROS)-induced damage and preserving skin-barrier integrity, thereby limiting inflammation-driven tissue injury within the pilosebaceous unit [23,24,25]. From a formulation perspective, CBD’s lipophilic nature facilitates its accumulation within the stratum corneum, enabling prolonged local activity with minimal systemic absorption following topical application. Permeation studies indicate that formulation vehicles such as propylene glycol and liquid paraffin can enhance dermal penetration, influencing therapeutic efficacy [20,29]. Clinical and preclinical investigations consistently report reductions in sebum production, inflammatory markers and acne lesion severity following topical application of CBD-containing formulations, with generally favourable safety and tolerability profiles [5,6].

Despite encouraging short-term outcomes, long-term safety data remain limited. Further well-designed clinical trials are required to establish standardised dosing parameters, formulation guidelines and comprehensive safety profiles to support the sustained clinical use of CBD in acne treatment [5,6].

### 3.2. Modulation of Inflammatory Signalling and Sebaceous Activity

Multiple in vitro and preclinical studies consistently report that hemp extracts and cannabidiol (CBD) significantly reduce pro-inflammatory cytokines, including tumour necrosis factor-alpha (TNF-α), interleukin-1 beta (IL-1β), interleukin-6 (IL-6), and interleukin-8 (IL-8), which are central mediators of acne-associated inflammation [16,20,25,30]. Suppression of these cytokines decreases inflammatory signalling within the pilosebaceous unit, leading to reduced sebocyte hyperactivity and a visible decrease in inflammatory acne lesions.

CBD exerts these effects primarily through modulation of the cutaneous endocannabinoid system (ECS). As illustrated in Figure S1, cannabinoid receptors CB_1_ and CB_2_ are widely distributed across epidermal keratinocytes, sebaceous glands, hair follicles, sensory nerves and sweat glands, enabling CBD to regulate multiple cellular processes relevant to acne pathogenesis. Acting on CB_1_ and CB_2_ receptors, transient receptor potential channels (TRPV1 and TRPV4) and peroxisome proliferator-activated receptor gamma (PPARγ), CBD modulates sebocyte proliferation, lipid synthesis, inflammatory mediator release and apoptosis, thereby restoring skin homeostasis [3,4,13,24,25].

Figure S1 demonstrates the distribution of endocannabinoids in the skin, highlighting the downstream functional outcomes of ECS activation, including reduced inflammation, decreased cellular proliferation, enhanced apoptosis and modulation of lipid synthesis, which collectively contribute to reductions in acne lesions. In parallel, CBD’s antioxidant properties mitigate oxidative stress within the pilosebaceous unit, limiting inflammation-driven tissue damage and lesion progression.

### 3.3. Antimicrobial Effects and Microbial Balance

Several studies demonstrate that hemp extracts and cannabidiol (CBD) inhibit *Cutibacterium acnes* biofilm formation and suppress *C. acnes*-induced inflammatory responses in vitro [16,20,25,30]. Clinical evidence also reports reductions in inflammatory lesion counts, although many formulations combine CBD with other active ingredients, making it challenging to attribute antimicrobial effects solely to CBD. Nevertheless, findings support a contributory role of hemp-derived compounds in modulating microbial dysbiosis and reducing inflammation-driven lesion progression [16,20,25,30]. Hemp extracts and cannabidiol (CBD) impairs biofilm development and bacterial proliferation, which helps minimise microbially driven irritation and lesion formation, thereby improving acne outcomes (Figure S2). Importantly, the antimicrobial activity of hemp-derived compounds offers a promising alternative to conventional antibiotics, potentially mitigating concerns related to antibiotic resistance [16,25,30]. Multiple antimicrobial evaluations have demonstrated that CBD oil effectively hinders the growth of *C. acnes*, a key bacterium implicated in acne pathogenesis, and disrupts biofilm formation critical for pathogenic colonisation and persistence within pilosebaceous units [25,31]. These effects collectively contribute to improved acne outcomes and position hemp-derived compounds as a viable adjunct or alternative to traditional antibiotic therapy [16,25,30,31].

### 3.4. Skin-Barrier Repair and Lipid Homeostasis

In addition to direct anti-inflammatory and antimicrobial effects observed with cannabinoid-containing hemp extracts, lipid-rich hemp seed-derived extracts may contribute indirectly to acne management by supporting epidermal barrier repair and lipid homeostasis. These extracts are characterised by a high proportion of omega-6 and omega-3 polyunsaturated fatty acids—notably, linoleic acid and α-linolenic acid, which are integral components of the stratum corneum lipid matrix [32,33,34].

Restoration of epidermal lipid organisation enhances barrier integrity, improves hydration and reduces transepidermal water loss, thereby limiting inflammatory flare-ups associated with barrier dysfunction [35,36]. Modulation of eicosanoid signalling pathways by polyunsaturated fatty acids may further contribute to attenuation of low-grade cutaneous inflammation relevant to acne pathophysiology [37,38].

Due to its lipophilic nature, cannabidiol (CBD) contained within cannabinoid-rich hemp extracts preferentially accumulates in the stratum corneum following topical application, enabling sustained local exposure with minimal systemic absorption. This characteristic supports a favourable cutaneous environment for barrier stability and anti-inflammatory signalling [6,23]. Both clinical and preclinical investigations report reductions in sebaceous activity, erythema and inflammatory lesions with generally favourable tolerability profiles [39]. However, frequent co-formulation of CBD with additional active ingredients remains a persistent confounding factor when attributing observed clinical effects to CBD alone.

### 3.5. Mechanistic Effects of Industrial Hemp Extracts on Acne Pathogenesis: Sebum Regulation and Skin-Barrier Homeostasis

Dysregulated sebum production is a central pathogenic feature of acne vulgaris, promoting follicular occlusion, microbial proliferation and downstream inflammation. Lipid-rich industrial hemp seed-derived extracts exert regulatory effects on sebaceous gland activity, predominantly through their distinctive fatty-acid composition and associated minor lipophilic constituents, which support epidermal barrier integrity and modulate inflammatory responses [3,4,23].

These extracts are particularly rich in polyunsaturated fatty acids, including omega-6 (linoleic acid) and omega-3 (α-linolenic acid), which play essential roles in cutaneous lipid metabolism. Linoleic acid deficiency has been linked to altered sebum composition and increased follicular hyperkeratinisation in acne-prone skin. Topical application of polyunsaturated-fatty-acid-rich hemp seed extracts may therefore help restore lipid balance, reduce excessive sebum secretion and limit comedone formation [3,15,20,27,40].

Importantly, the balanced omega-6-to-omega-3 ratio characteristic of hemp seed-derived lipid extracts supports anti-inflammatory signalling pathways that complement sebostatic effects. Unlike highly occlusive lipid preparations, these extracts are typically non-comedogenic, allowing for modulation of sebum output without exacerbating follicular blockage.

Beyond sebum regulation, lipid-rich hemp extracts contribute to maintenance of the hydrolipidic barrier by improving moisture retention and supporting epidermal homeostasis. This dual action normalising sebum flow while preserving hydration may help prevent cycles of sebaceous overactivity and compensatory dryness that perpetuate acne flare-ups [15,20,27,40]. Minor bioactive constituents present in hemp seed extracts may further act synergistically to enhance barrier repair and anti-inflammatory responses, reinforcing skin resilience.

Collectively, these properties position lipid-rich hemp seed-derived extracts as supportive dermocosmetic ingredients in integrative acne management strategies, acting primarily through barrier and lipid modulation rather than direct cannabinoid-mediated effects [20,27].

### 3.6. Cutibacterium acnes Suppression

Multiple antimicrobial evaluations have demonstrated that hemp oil effectively hinders the growth of *Cutibacterium acnes*, a key bacterium implicated in acne pathogenesis [25,31]. This inhibition extends to the disruption of the formation of bacterial biofilms, which are critical for pathogenic colonisation and persistence within the pilosebaceous units [31] (Figure S2). In impairing biofilm development and bacterial proliferation, hemp oil reduces microbially driven irritation and lesion formation, thereby contributing to improved acne outcomes. The antimicrobial activity of hemp-derived compounds offers a promising alternative to conventional antibiotics, potentially mitigating concerns related to antibiotic resistance [16,25,30].

### 3.7. Skin Inflammation Mitigation

Empirical evidence indicates that topical application of hemp oil significantly reduces erythema and inflammatory biomarkers in the skin, reinforcing its role as a potent anti-inflammatory cosmeceutical for acne and other dermatological conditions [3,13,41]. The bioactive constituents of hemp oil modulate key inflammatory mediators, including cytokines such as TNF-α and IL-1β, thereby alleviating local inflammation and associated symptoms such as redness and swelling [3,13,42]. This anti-inflammatory potential further underscore hemp oil’s therapeutic value in skincare formulations designed to manage inflammatory skin disorders [3,13,41,42].

### 3.8. Cosmeceutical Integration and Delivery Optimisation

Industrial hemp-derived compounds align with a cosmeceutical and nutraceutical approach to acne management, offering multi-targeted efficacy with fewer adverse effects than conventional pharmacological treatments such as retinoids, antibiotics and isotretinoin, which are commonly associated with irritation, photosensitivity and antibiotic resistance [29,43].

Despite promising bioactivity, the clinical efficacy of topical CBD is limited by poor aqueous solubility, physicochemical instability and variable dermal penetration. To overcome these barriers, nanotechnology-based delivery systems including liposomes, nanostructured lipid carriers (NLCs), nano emulsions, cryogels, microneedles, transdermal patches and inorganic nanoparticles have been developed to enhance stability, bioavailability and targeted skin delivery [14,24,40,44].

Experimental evidence indicates that nano-encapsulated CBD exhibits superior pharmacokinetics, enhanced anti-inflammatory efficacy, improved dermal penetration and reduced irritation compared with conventional formulations. Table 3 depicts that inorganic CBD-capped nanoparticles, such as gold- or zinc oxide-based systems further demonstrate synergistic anti-inflammatory and antimicrobial effects [13,16,25,30,43,44]. A pilot randomised clinical study reported that nano-encapsulated CBD cream mitigated UV-A-induced nuclear and mitochondrial DNA injury, supporting enhanced topical performance [8]. However, most supporting evidence remains preclinical or early-phase, and regulatory approval for topical CBD formulations is still limited relative to oral products such as Epidiolex^®^ manufactured by Jazz Pharmaceuticals Research UK Limited located in Oxford United Kingdom. [45,46].

### 3.9. Phytochemical Synergy, Sustainability and Evidence Gaps

Table 4 illustrates the substantial phytochemical diversity of industrial hemp extracts, which include cannabinoids (CBD, CBG, CBC and THCV), terpenes, polyphenols, lignanamides and essential fatty acids, each contributing complementary dermatological functions through distinct biological pathways [23,47,48,49]. Cannabinoid-containing extracts derived from flowers and aerial parts are primarily associated with sebostatic, anti-inflammatory and immunomodulatory effects, whereas non-cannabinoid constituents such as terpenes and polyphenols contribute antioxidant and antimicrobial activities.

Lipid-rich seed-derived hemp extracts (often referred to as hemp seed oil) are characterised by high levels of polyunsaturated fatty acids and contribute primarily to skin-barrier support and hydration rather than direct endocannabinoid signalling. While whole-plant extracts may theoretically exert synergistic or “entourage-like” effects, substantial heterogeneity in cultivation, extraction methods and chemical standardisation remains a major barrier to reproducibility, regulatory approval and clinical translation [3,4,50].

### 3.10. Contribution of Fatty Acid-Dominant Hemp Seed-Derived Extracts

The fatty-acid profile of lipid-rich hemp seed-derived extracts plays a supportive role in maintaining sebum balance and epidermal barrier function, both of which are relevant to acne-prone skin [19,51,52,53,54]. These extracts are particularly rich in omega-6 and omega-3 polyunsaturated fatty acids, which influence cutaneous lipid metabolism and sebum composition rather than directly suppressing sebocyte activity through cannabinoid receptor pathways.

A balanced omega 3 and 6 fatty acids ratio may contribute to attenuation of inflammatory signalling and support restoration of the stratum corneum lipid matrix, potentially reducing follicular hyperkeratinisation and secondary inflammatory flare-ups [51,54]. These barrier-supportive effects operate independently of the endocannabinoid system and should be interpreted as adjunctive rather than therapeutic mechanisms in acne management. Figure S3 depicts a schematic illustration of lipid-rich hemp seed-derived extract penetration, modulation of barrier lipids and support of non-comedogenic sebum flow.

### 3.11. Topical Application Approaches

Topical application of cannabinoid-containing hemp extracts exerts biological effects through modulation of the cutaneous endocannabinoid system. Cannabidiol (CBD) interacts with CB_1_, CB_2_, TRPV1, TRPV4 and PPARγ receptors, regulating inflammatory cascades, sebocyte proliferation and lipid synthesis within the pilosebaceous unit [18,19,51]. These receptor-mediated pathways underpin the sebostatic and anti-inflammatory effects reported in preclinical and early clinical acne studies.

In contrast, lipid-rich hemp seed-derived extracts do not directly activate ECS receptors but contribute to epidermal homeostasis by reinforcing the hydrolipidic barrier, enhancing moisture retention and reducing transepidermal water loss [3,19,20,50]. Clear mechanistic separation of these pathways is essential, as over-attribution of ECS effects to fatty acid-dominant extracts represents a common source of confusion in the literature. Figure S4 shows the molecular pathway diagram, highlighting ECS receptor targets and downstream effects specific to cannabinoid-containing hemp extracts.

### 3.12. Safety, Regulation, Evidence Gaps and Grading

Across available studies, the evidence indicates that industrial hemp oil and CBD-based dermatological products generally exhibit favourable short-term safety profiles when applied topically, with most reported adverse effects being mild and transient, such as minimal skin irritation or erythema [24,55,56]. These findings support their potential suitability for cosmetic and cosmeceutical applications targeting acne-prone skin. Despite encouraging short-term outcomes, long-term safety and efficacy remain unverified, and data in vulnerable populations, including pregnant individuals, paediatric patients and immunocompromised groups, are notably lacking. Consequently, caution is warranted when extrapolating short-term findings to prolonged or widespread clinical use [24,56].

Quality inconsistencies, contamination risks and fragmented regulatory frameworks further underscore the need for harmonised standards governing extraction, formulation, dosing and clinical evaluation to ensure safe and effective dermatological applications [24,49,55,56]. Table 5 illustrates evidence grading aligned with risk-of-bias tools reported in the Methods (Cochrane RoB 2 for clinical trials and SYRCLE for preclinical studies) and the consistency of outcomes across models. In vitro keratinocyte and fibroblast studies indicate that low concentrations of cannabidiol (CBD) exert antioxidant and pro-repair effects, including modulation of TGF-β, VEGF and NF-κB signalling pathways and upregulation of collagen-related gene expression, suggesting potential support for skin barrier recovery; certainty remains low, pending clinical confirmation [7,8,9] To address these gaps, well-designed long-term clinical trials are required to establish standardised dosing parameters, formulation guidelines and comprehensive safety profiles that support the sustained clinical use of CBD in acne treatment.

### 3.13. Sustainability in Hemp Production

Hemp cultivation is widely recognised as a highly sustainable agricultural practice with multiple environmental benefits. Compared to conventional crops such as cotton, hemp requires significantly less water, making it suitable for regions experiencing water scarcity [41,53]. Its deep root system improves soil structure by aerating compacted soils, reducing erosion and enhancing moisture retention, which supports long-term soil health and fertility [54,58]. Additionally, hemp serves as an effective carbon sink, capturing and storing more atmospheric carbon dioxide per hectare than many other crops [52,58]. This carbon sequestration capacity, combined with its rapid growth cycle, positions hemp as a valuable contributor to climate change mitigation by lowering net greenhouse gas emissions [53].

The crop’s low reliance on pesticides and chemical fertilisers further reduces its environmental footprint, limiting chemical overflow and ecosystem pollution [41,54]. Hemp farming also promotes biodiversity by creating habitats for pollinators and beneficial insects while reducing harmful agrochemical exposure [57]. Its versatility as a renewable raw material for textiles, paper, biofuels and construction materials alleviates pressure on forest resources, potentially curbing deforestation rates [53,57]. Life-cycle assessments confirm that organic and outdoor cultivation methods deliver the lowest environmental impacts across carbon footprint, water use, acidification and eutrophication categories, whereas indoor cultivation exhibits comparatively higher footprints [53].

Sustainability considerations extend to supply-chain resilience, organic certification, traceability and regulatory fragmentation, which remain significant challenges for industrial hemp-based cosmeceuticals [17,24,49,59]. Addressing these gaps requires harmonised global standards governing extraction, formulation, dosing and clinical evaluation to ensure safe and effective applications [49,55]. Importantly, sustainable hemp production also supports economic and social dimensions by enabling efficient land use, strengthening local economies, promoting safer working conditions and aligning with circular economy principles through full utilisation of hemp biomass [54,58].

### 3.14. Resilience of the Industrial Hemp Supply Chain

The industrial hemp supply chain is demonstrating increasing resilience in response to evolving regulatory, economic and environmental challenges projected for 2025 and beyond [42,60]. Despite recent global production fluctuations and downward revisions in natural fibre volumes, stakeholders are adapting through enhanced traceability, certification standards and strategic sourcing diversification to mitigate risks associated with supply inconsistencies and price volatility [42,60]. The Responsible Hemp Standard (RHS) exemplifies these efforts through rigorous documentation and third-party audits that ensure transparency and product authenticity, strengthening consumer trust in a fragmented market [60].

Leading companies are embracing vertical integration and technological innovation to secure stability across cultivation, processing and manufacturing stages. Investments in modular processing facilities and flexible extraction technologies enable rapid adjustments to meet raw material availability and evolving product demand [60]. Collaborative partnerships between seed breeders, processors and end-user industries such as the textile, automotive and construction industries further diversify product streams, enhancing supply-chain adaptability [42].

Sustainability initiatives, including water-efficient irrigation systems, renewable energy adoption and zero-waste production models, reduce environmental footprints while reinforcing supply-chain robustness [42,60]. Digital solutions such as blockchain-based traceability platforms provide real-time visibility into origin and processing data, which is critical for compliance and meeting consumer expectations [60].

These strategies align with SDG 12 (Responsible Consumption and Production), promoting sustainable supply chains, resource efficiency and transparency in production systems. Through environmental stewardship, technological innovation and regulatory compliance, the industrial hemp sector contributes to global sustainability goals while ensuring long-term market resilience.

## 4. Discussion

Mechanistic findings consistently demonstrate attenuation of key pro-inflammatory cytokines, including TNF-α, IL-1β, IL-6 and IL-8, with associated modulation of sebocyte activity in vitro, supporting biological plausibility, given established endocannabinoid signalling in the skin [20,21,22,23,61].

In grading the evidence, we considered reported risk-of-bias assessments (CochraneRoB-2) for clinical trials and SYRCLE adaptations for preclinical work and the consistency of outcomes across models. Anti-inflammatory and sebostatic actions of CBD and hemp extracts are supported with moderate certainty due to reproducible reductions in pro-inflammatory cytokines and lipogenesis across independent in vitro models and limited ex vivo data [20,21,22,23,61]. Clinical efficacy in terms of lesion counts and erythema is graded with low certainty because sample sizes are small, randomised controlled trials are absent and co-formulation with other actives confounds attribution [5,6]. Antimicrobial activity against *C. acnes* is rated with low certainty, as the evidence base is largely in vitro, with limited clinical endpoints [46,56,59]. Formulation strategies such as nano-encapsulation, nanoemulsions and the use of permeation enhancers may improve cannabidiol (CBD) stability and cutaneous penetration; however, dermatology-focused clinical trials remain limited, and reported endpoints are frequently non-acne-specific [9,13,14,24]. Short-term topical safety is rated with low to moderate certainty, given consistent reporting of mild irritation and a lack of serious adverse events in available trials and observational reports [6,39]. These grades align with the summary in the Section 2, which notes elevated bias risk of approximately 40% (high risk) in trials due to blinding limitations and 60% (high risk) in preclinical settings using SYRCLE, reflecting heterogeneity in formulations, doses and outcome measures across the literature.

Interpretation is limited by several sources of heterogeneity. Designs vary widely, with non-standardised CBD concentrations vehicles and treatment schedules, which complicates dose–response analysis [5,6]. Outcomes range from biochemical cytokine assays to lesion counts, without uniform severity scales or patient-reported measures, which restricts comparability [20,39,43]. Many clinical products combine CBD with other actives, such as salicylic acid, silymarin and Centella asiatica; hence, observed benefits cannot be attributed to CBD or hemp oil alone [5,24].

Besides clinical performance, hemp offers sustainability attributes that are relevant to dermatological product development. Life-cycle assessments and agronomic studies indicate lower water requirements than many comparator crops, improved soil structure through deep rooting and meaningful carbon sequestration per hectare [31,52,53,54,57]. Reduced reliance on pesticides, coupled with habitat support for pollinators, further decreases environmental burden and aligns with clean-label cosmetic expectations [41,54]. At a supply-chain level, resilience strategies include certification and traceability frameworks such as the Responsible Hemp Standard and investments in modular processing and diversified sourcing to buffer raw-material volatility [42,60]. To translate these advantages into cosmeceutical practice, sustainability metrics should be reported alongside efficacy. We recommend disclosure of the cultivation method (organic versus conventional extraction processes) and energy inputs (water footprint, carbon balance, packaging footprint and end-of-life plans for material) [18,31,53]. Embedding these indicators within product dossiers and regulatory submissions enables differentiation of hemp-based cosmeceuticals while improving transparency and consumer trust. Practical measures include formulation transparency, with CBD concentration and co-actives clearly labelled, together with sustainability certifications where applicable, adoption of cradle-to-grave life-cycle assessment consistent with ISO 14040 and eco-design packaging that uses recyclable or biodegradable materials to complement the sustainability narrative of hemp-derived actives [62].

From a clinical standpoint, hemp-derived cosmeceuticals containing defined concentrations of cannabidiol (CBD) may be positioned as adjunctive interventions for mild-to-moderate inflammatory acne, with emphasis on anti-inflammatory and sebostatic mechanisms rather than definitive antimicrobial replacement therapy [5,6,23,39]. Transparent labelling of CBD content, vehicle formulation and disclosure of co-active ingredients are essential to ensure clinical interpretability, reproducibility and appropriate patient counselling [5,57].

Available evidence indicates that short-term topical tolerance of CBD-containing formulations is generally favourable, with most reported adverse events limited to mild and transient irritation or erythema. However, long-term safety data across diverse populations remain limited, necessitating clinical monitoring for irritation, photosensitivity and interactions with concurrent topical or systemic therapies [6,24,39,56].

Emerging evidence related to the gut–skin axis provides a complementary framework for understanding how systemic inflammation and barrier function may be influenced by diet and nutritional inputs. Oral hemp-derived products such as seeds and fermented hemp beverages introduce prebiotic fibres, essential fatty acids, proteins and probiotic microorganisms, which may indirectly modulate systemic inflammatory tone and cutaneous immune balance [7,8,22]. These nutritional effects should be interpreted as supportive and adjunctive and are mechanistically distinct from the topical ECS-mediated actions of CBD [21,23].

Integration of topical cannabinoid-containing formulations with broader lifestyle and nutritional approaches within sustainability-anchored hemp supply chains offers a holistic and practice-relevant model for advancing skin health while reinforcing responsible production and consumption principles [31,42,53,60].

## 5. Research Priorities and Evidence Gaps

Key research priorities include randomised controlled trials employing concealed allocation that compare monotherapy CBD formulations with placebo or standard-of-care treatments using validated acne severity indices and patient-reported outcomes [5,6]. Dose-ranging and formulation-comparative studies are required to define therapeutic windows across delivery systems, including gels, emulsions and nanocarriers, with standardised reporting of concentration, application frequency and treatment duration [13,14,24,44,45].

Comprehensive safety evaluation should extend beyond short-term tolerability and include patch testing, phototoxicity assessments and long-term follow-up, with stratification by age, skin phototype and comorbid dermatological conditions [8,24,39,56]. Mechanistic investigations in human skin tissue and ex vivo models are required to confirm pathway modulation observed in cell lines, including direct assessment of sebaceous activity, inflammatory markers and ECS receptor expression through biopsy-based and advanced imaging techniques [20,23,61].

Sustainability and transparency should be embedded within future trials and product dossiers through life-cycle assessment, traceability audits and recognised certification frameworks, enabling alignment of clinical benefit with demonstrable reductions in environmental footprint and supply-chain risk [6,31,42,53,60].

## 6. Conclusions

This systematic review highlights the multifaceted value of industrial hemp (*Cannabis sativa* L.) within sustainable and resilient supply chains and identifies cannabinoid-containing hemp extracts, particularly cannabidiol (CBD), as the bioactive components of the greatest relevance for cosmeceutical applications in acne management [6,21,22]. Across preclinical and early clinical investigations, CBD-containing formulations demonstrate anti-inflammatory and sebostatic activity, with advances in nano- and carrier-based delivery systems offering potential improvements in topical stability and cutaneous bioavailability [6,22,23].

Lipid-rich hemp seed-derived products and hemp-based foods were considered only in a contextual capacity, as sources of essential fatty acids and nutritional components that may support epidermal barrier integrity and systemic inflammatory balance. These effects act primarily through nutritional and barrier-supportive pathways rather than direct endocannabinoid system modulation and should not be interpreted as therapeutic substitutes for cannabinoid-defined interventions [6,21,22]. Incorporation of gut–skin axis concepts provides a complementary framework for understanding dietary and metabolic influences on skin homeostasis; however, such mechanisms remain adjunctive and insufficiently characterised in acne-specific clinical studies [21,22].

Beyond biological efficacy, this review emphasises the importance of supply-chain standardisation, regulatory harmonisation and traceability to ensure product quality, consumer trust and reproducibility of clinical outcomes. Harmonised reporting of cultivar origin, extraction methods, cannabinoid composition and sustainability metrics is essential to support regulatory evaluation and to enable meaningful comparison across studies [6,31,42,53,60].

While the current evidence positions CBD-containing hemp extracts as promising adjunctive cosmeceuticals for inflammatory acne, antimicrobial effects and long-term clinical benefit remain tentative. Future progress in this field will depend on methodologically rigorous clinical investigations, including high-quality non-randomised and controlled studies using standardised formulations, validated acne severity outcomes and comprehensive safety assessment, with advancement toward comparative and randomised designs where feasible [6,56].

Collectively, the available evidence supports the biological plausibility and emerging clinical relevance of CBD-containing hemp extracts in acne management while positioning hemp-derived lipid-rich and nutritional products as supportive contributors through barrier-related and systemic pathways rather than direct therapeutic effects. Integration of sustainability considerations into study design, regulatory evaluation and product development will be central to guiding responsible innovation and supporting credible clinical translation of hemp-derived cosmeceuticals in acne care [6,31,42,53,60].

## Figures and Tables

**Table 1:** Characteristics of key mechanistic, clinical and delivery-system studies included for evidence synthesis or contextual interpretation.

Reference	Model or Population	Formulation/Intervention	CBD Concentration or Dose	Duration/Exposure	Key Outcomes	Confounders/Notes
[1]	In vitro human sebocytes and human skin organ culture	Purified CBD	1–10 µM	Acute and repeated exposure	↓ Lipogenesis, ↓ sebocyte proliferation, ↓ inflammatory signalling (NF-κB/MAPK); TRPV4 and A2A involvement	Mechanistic study; no clinical endpoints
[2]	Human epidermal keratinocytes + *C. acnes* extracellular vesicles	CBD	0.5–2 µM	Acute exposure	↓ IL-6, IL-8, TNF-α; CB2 upregulation; NF-κB/MAPK inhibition	In vitro inflammatory model; supports mechanistic plausibility
[7]	Adults with mild–moderate acne	Gel containing CBD + Centella asiatica triterpenes, silymarin, salicylic acid	CBD 1% (co-formulated)	56 days	↓ Inflammatory lesion counts	Multi-active formulation; attribution to CBD alone not possible
[10]	Healthy human volunteers (pilot RCT)	Nano-encapsulated CBD cream	1% CBD (topical)	Short-term application with UV-A exposure	↓ Nuclear and mitochondrial DNA damage	Non-acne endpoint; supports topical delivery performance

Abbreviations: ↓ refers to decreased.

**Table 2:** Risk-of-bias assessment for included clinical studies.

Reference	Design	Randomisation	Deviations	Missing Data	Outcome Measurement	Reported Result
[7]	Non-randomised (ROBINS-I)	N/A	Low risk	Low risk	Some concerns	Some concerns
[8]	Non-randomised (ROBINS-I)	N/A	Some concerns	Low risk	Some concerns	ROBINS-I: serious risk (overall)

N/A (not applicable).

**Table 3:** Nano-formulated systems.

Nano-Formulation Type	Mechanism	Observed Benefit	Reference
Nanostructured Lipid Carrier (NLC) Gel	Co-loaded lipid system	Sustained release, reduced irritation	[15]
Liposomal CBD	Encapsulation within phospholipid bilayers	Enhanced skin penetration and hydration	[18]
Hybrid Nano-Emulsions	Colloidal hybrid stabilisers	Improved epidermal permeation and wound healing	[18]
CBD-Capped Inorganic Nanoparticles	Metallic nanoparticle surface modification	Synergistic anti-inflammatory and antimicrobial effects	[42]

**Table 4:** Phytochemical diversity of industrial hemp extracts and dermatological relevance.

Compound Class	Key Examples	Reported Function	Evidence Strength
Cannabinoids	CBD, CBG, CBC, THCV	Anti-inflammatory, sebostatic	High (clinical and preclinical)
Terpenes	β-caryophyllene, limonene	Antioxidant, antimicrobial	Moderate
Polyphenols	Cannflavin A, lignanamides	Antioxidant, anti-inflammatory	Moderate
Fatty Acids	Linoleic, α-linolenic acids	Skin-barrier repair, hydration	High

**Table 5:** Evidence grading aligned with risk-of-bias tools.

Domain	Summary of Evidence	Certainty
Anti-inflammatory and sebostatic mechanisms (CBD and hemp extracts)	Reproducible cytokine reductions (TNF-α, IL-1β, IL-6 and IL-8) and lipogenesis suppression across independent in vitro models, with limited ex vivo support [1,2,6,21,24]	Moderate
Clinical efficacy (lesion counts and erythema)	Small samples, absence of RCTs and frequent co-formulation with other actives [7,8]	Low
Short-term topical safety	Mild local irritation under ten percent and no serious adverse events across reports [7,8]	Low to moderate
Antimicrobial activity against *C. acnes*	Predominantly in vitro evidence and limited clinical endpoints [52,55,57]	Low

## Data Availability

The original contributions presented in this study are included in the article/Supplementary Material. Further inquiries can be directed to the corresponding author.

## Supplementary Materials

The following supporting information can be downloaded at: https://www.mdpi.com/article/10.3390/molecules31122017/s1, Table S1: Full-text studies excluded after eligibility assessment and reasons for exclusion [57,63]. Figure S1: Distribution of endocannabinoids in the skin. Figure S2: Acne pathogenesis. Increased sebum production, accumulation of *C. acnes*, hyperkeratinisation and inflammation resulting in the formation of comedones. Figure S3: A Schematic illustration of lipid-rich hemp seed-derived extract penetration, modulation of barrier lipids and support of non-comedogenic sebum flow. Figure S4: Molecular pathway diagram highlighting ECS receptor targets and downstream effects specific to cannabinoid-containing hemp extracts.

## Abbreviations

The following abbreviations are used in this manuscript:

CBD	Cannabidiol
THC	Tetrahydrocannabinol
CB_1_	Cannabinoid Receptor Type 1
CB_2_	Cannabinoid Receptor Type 2
TRPV1	Transient Receptor Potential Vanilloid 1
TRPV4	Transient Receptor Potential Vanilloid 4
TNF-α	Tumour Necrosis Factor Alpha
IL-1β	Interleukin 1 Beta
IL-6	Interleukin 6
IL-8	Interleukin 8
PPARγ	Peroxisome Proliferator-Activated Receptor Gamma
ECS	Endocannabinoid System
NF-κB	Nuclear Factor Kappa-Light-Chain-Enhancer of Activated B Cells
MAPK	Mitogen-Activated Protein Kinase
RCT	Randomised Controlled Trial
PRISMA	Preferred Reporting Items for Systematic Reviews and Meta-Analyses
SDG	Sustainable Development Goal
NLC	Nanostructured Lipid Carrier
PROSPERO	International prospective register of systematic reviews
ROS	Reactive Oxygen Species
PUFAs	Polyunsaturated Fatty Acids
RHS	Responsible Hemp Standard
WHO	World Health Organization
BMJ	British Medical Journal
PLoS ONE	Public Library of Science ONE
