# Supplementary material for: CBD-Containing Hemp Extracts and Isolated CBD for Acne: A Systematic Review of Anti-Inflammatory Mechanisms, Clinical Signals and Sustainability
Source: Molecules. 2026 Jun 9;31(12):2017. doi: 10.3390/molecules31122017 (PMC13304674; doi:10.3390/molecules31122017)
Supplement: Supplementary file 1 [file molecules-31-02017-s001.zip › molecules-4091608-supplementary.pdf]

molecules

MDPI

Supplementary Table S1. Full-text studies excluded after eligibility assessment and reasons for exclusion

Citation	Reason for exclusion	Notes
Thammasat University, 2021	Hemp seed extract; no quantified cannabinoids	Split-face cosmetic study; outside CBD efficacy scope
MDPI Cosmetics, 2025	Non-CBD hemp seed extract; skincare endpoint only	Oxygenating mask; no acne or cannabinoid analysis

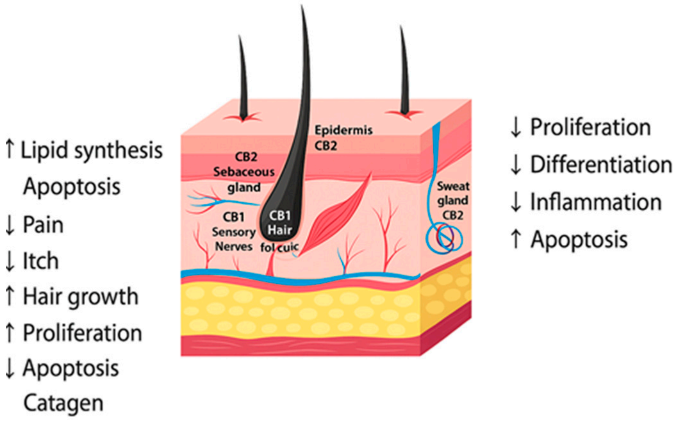

Supplementary Figure S1. Distribution of endocannabinoids in the skin

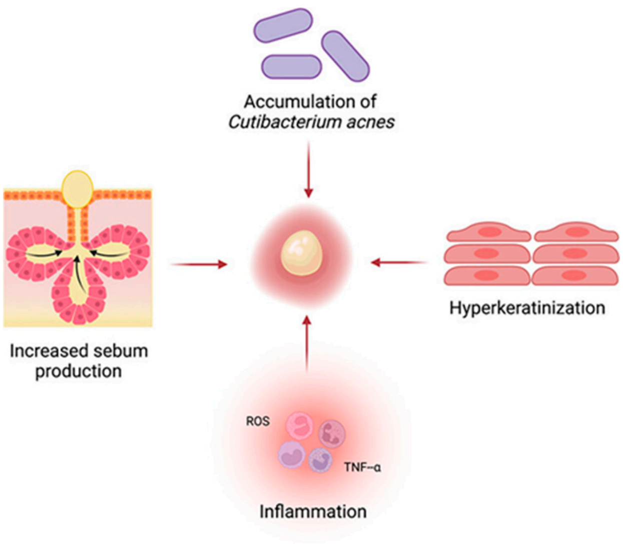

Supplementary Figure S2. Acne pathogenesis. Increased sebum production, accumulation of C. acnes, hyperkeratinisation and inflammation resulting in the formation of comedones.

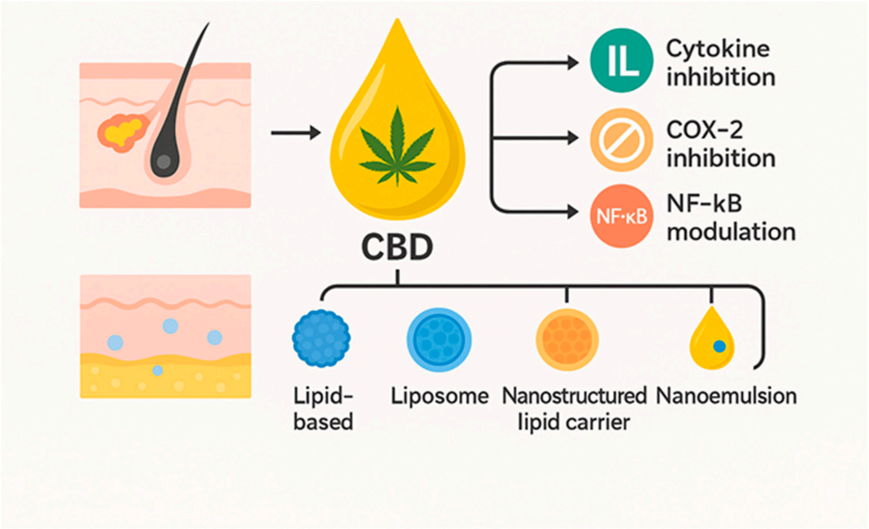

Supplementary Figure S3. A Schematic illustration of lipid-rich hemp seed–derived extract penetration, modulation of barrier lipids and support of non-comedogenic sebum flow.

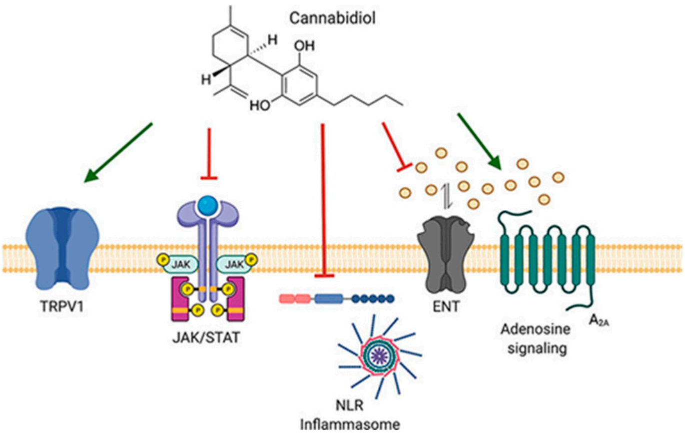

Supplementary Figure S4. Molecular pathway diagram highlighting ECS receptor targets and down-stream effects specific to cannabinoid-containing hemp extracts.
